# Explain the behavior change and maintenance in diabetic patients using MTM-HAPA framework

**DOI:** 10.3389/fpsyt.2024.1497872

**Published:** 2024-12-09

**Authors:** Yibo Wu, Zhenjie Yu, Xiaoqiu Yin, Yimiao Li, Yang Jiang, Gongli Liu, Xinying Sun

**Affiliations:** ^1^ Department of Social Medicine and Health Education, School of Public Health, Peking University, Beijing, China; ^2^ Department of Infectious Diseases and Public Health, Jockey Club College of Veterinary Medicine and Life Sciences, City University of Hong Kong, Hong Kong, Hong Kong SAR, China; ^3^ Institute for Advanced Studies in Humanities and Social Sciences, Beihang University, Beijing, China; ^4^ School of Nursing, Tianjin Medical University, Tianjin, China; ^5^ Jitang College, North China University of Science and Technology, Tangshan, Hebei, China; ^6^ Department of Epidemiology and Health Statistics, School of Public Health, Qingdao University, Qingdao, China

**Keywords:** health behavior, diabetes mellitus, multi-theory model of health behavior change, health action process approach, psychology

## Abstract

**Objectives:**

The aim of the study was to to uncover the factors influencing the initiation and maintenance of health behaviors indiabetes mellitus (DM) patients, utilizing baseline data from a randomized controlled trial to construct a structural equation model based on the Multi-Theory Model (MTM) and Health Action Process Approach (HAPA) scales.

**Methods:**

The study recruited participants with type 2 diabetes, aged between 18 and 75 years, from 45 distinct locations in Beijing, China.Patients [N = 406, n = 232 (57.1%) females, n = 232 (42.9%) males; Mean (SD) age = 56.7(10.9)] completed self-reported questionnaire about constructs from integrated theories concerning health behavior. To test the associations between the variables, structural equation modeling with latent variables was employed. Based on the path coefficients of Structural Equation Modeling(SEM), we verified all the hypotheses.

**Results:**

Disadvantages, Advantages, Self-efficacy for Initiating Behavior, and Changes in Physical Environment are all prove to have an effect on intention, with the effect of Disadvantages being negative. Intention positively influenced Action Planning and Coping Planning, both of which in turn significantly predicted Initiation of Behavior Change. Practice for change, Emotional Transformation, Changes in Social Environment, and Self-efficacy for Sustaining Behavior were all affected by Outcome Expectancies and Risk Perception positively. Meanwhile, Practice for change, Emotional Transformation, Changes in Social Environment and Self-efficacy for Sustaining Behavior- would have a significant predictive effect on Maintenance of Behavioral Change.

**Conclusion:**

The empirical evidence from this study robustly validates the majority of its theoretical constructs, affirming that MTM-HAPA possess significant explanatory capability in delineating the factors that underpin both the Maintenance of health-related behaviors and the Initiation of Behavior Changes in individuals suffering from DM.

## 1 Introduction

Diabetes Mellitus (DM) is a multifaceted clinical syndrome that arises from a combination of genetic predispositions and environmental influences. It is characterized by an absolute or relative deficiency in insulin secretion, as well as a diminished cellular response to insulin, which leads to various metabolic disruptions (1). According to the 10th edition report of the International Diabetes Federation (IDF), the global challenge of diabetes is intensifying, with the number of affected adults aged 20-79 reaching 537 million in 2021. This figure is projected to increase to 643 million by 2030. Particularly in China, the prevalence among this age group was recorded at 140 million in 2021, and is expected to surge to 174 million by 2045 (2). The persistent rise in diabetes cases over the past three decades highlights its growing impact on public health and its substantial economic toll on societies worldwide. In 2021 alone, the global healthcare expenditures associated with diabetes neared one trillion US dollars, with China bearing a significant portion of these costs, approximately 16.5 billion US dollars (3).

Diabetes is a chronic condition that, while manageable, remains incurable (4). It is often accompanied by a variety of long-term complications, such as diabetic retinopathy, nephropathy, neuropathy, and diabetic foot disease (5). These complications not only pose a significant challenge to patients in implementing effective blood sugar management, but also severely impact their mental health, often leading to emotions (6) such as depression, sadness, and irritability. These psychological burdens (7) not only weaken patients’ ability to interact socially (8), reducing the quality of their daily lives (9), but may also diminish their willingness to follow treatment plans, thereby hindering effective blood sugar control (10, 11) and the overall progression of the disease (12, 13). Studies have shown that people with diabetes are twice as likely to suffer from depression as the general population, and psychological distress is closely related to difficulties in self-managing diabetes and controlling the disease (14, 15). Therefore, numerous research projects are dedicated to improving the health behaviors of people with diabetes, with the aim of enhancing their long-term health outcomes (16). Nonetheless, sustaining these behavior changes post-discharge remains a significant hurdle, with patients often reverting to their initial behavior patterns within months (17). Thus, developing innovative approaches to reshape patients’ health beliefs, promoting sustained behavior modifications, and supporting the adoption of healthy, sustainable lifestyles are essential (18). Enhancing patient self-efficacy and fostering enduring health behaviors are now paramount public health goals that require national and international attention.

The Multi-Theory Model (MTM) is an emerging behavioral theory model designed by Sharma in 2015. Since the advent of MTM, researchers from various countries have validated it in different populations (19), focusing mainly on exercise, healthy eating, substance addiction management, mental health, and medical adherence. Their research has yielded good results, indicating that MTM can be used for different populations, but its effectiveness for diabetes remains unknown. However, there are still some limitations to using these theories to explain the key factors of behavioral variables. Most MTM studies have focused on the initiation and maintenance stages of exercise behavior, with few studies assessing the impact of the behavioral volition stage. It is worth noting that health behavior change is a behavior that requires persistence, and determining the factors that affect the maintenance of health behavior is important. Therefore, it is necessary to develop relevant behavioral theories to guide the behavior change and maintenance of diabetic patients. The Health Action Process Approach (HAPA) is a new health behavior stage theory proposed by Schwarzer. It has been used in many health behavior studies to determine the psychological determinants of health behavior. The theory includes the motivation phase and the volition phase. In the motivation phase, an individual’s intention to perform a certain behavior is influenced by task self-efficacy (an optimistic belief), outcome expectations (whether a person believes that a certain behavior will lead to desired changes), and risk perception (the perceived health threat or the concern that needs to mobilize action). The volition phase includes action plans specifying when, where, and how to perform the behavior, and coping plans to avoid anticipated obstacles. The HAPA model shows that behavior depends not only on action and coping plans but also on the perceived ability to persist in behavior (self-efficacy for sustaining behavior) and to cope after ending the behavior (recovery self-efficacy). Therefore, this study takes the MTM structure as the main model, combining HAPA’s task self-efficacy and self-efficacy for sustaining behavior with MTM’s self-efficacy for Initiating behavior and confidence in maintaining behavior, and applies it to diabetic patients. This study integrates MTM and HAPA to form the MTM-HAPA model. In order to explore the factors affecting the initiation and maintenance of health behaviors in diabetic patients based on the integrated model, and to provide a theoretical basis for the development of multi-dimensional, personalized interventions. The main hypotheses are as follows:

During the initial stage of behavior initiation, the advantages of behavior change, changes in the physical environment, and self-efficacy for Initiating behavior are positively correlated with behavioral intention(H1, H3,H4). The disadvantages of behavior change are negatively correlated with behavioral intention(H2). The disadvantages of behavior change are negatively correlated with self-efficacy for Initiating behavior(H5).Behavioral intention is positively correlated with action plans and coping plans(H6,H7). Action plans and coping plans are positively correlated with the initiation of behavior change(H8,H9). In the maintenance stage of behavior initiation, risk perception, outcome expectations are positively correlated with self-efficacy for sustaining behavior, changes in the social environment, practice for change, and emotional transformation(H10-H17). self-efficacy for sustaining behavior, changes in the social environment, practice for change, and emotional transformation are positively correlated with the maintenance of behavior change(H18-H21).

## 2 Theoretical basis

Health behavior encompasses proactive strategies adopted by individuals to ward off diseases and maintain optimal health (20). These strategies include altering detrimental lifestyles such as smoking, excessive alcohol consumption, poor dietary habits, and engaging in unprotected sexual activities. Conversely, it involves cultivating beneficial practices like regular physical exercise, frequent health check-ups, and adherence to medical advice (21). In the realm of health education and promotion, Sharma’s exploration of multigenerational health behavior improvement programs reveals that despite the existence of various models aiming to enhance health behaviors, the complexity of factors influencing these behaviors meant that the initial three generations of models did not provide a comprehensive explanation (22). The latest, fourth-generation Multi-Theory Model (MTM) offers a more detailed analytical framework by amalgamating multiple theories, addressing both the initiation and maintenance of health behaviors through a layered conceptual approach, aiming to foster long-term changes (23).

The MTM is bifurcated into two main components: initiation and maintenance of behavior change. Sharma highlights the necessity of distinguishing between these phases since the factors influencing them differ significantly, and previous models lacked the specificity to separately predict these dynamics. For initiating behavior changes, the MTM identifies three pivotal factors: participatory dialogues, behavior confidence, and modifications in the physical environment. For maintaining behavior changes, it emphasizes emotional transformation, practice alterations, and shifts in the social environment (24). Although these components are related and demonstrate some consistency across the stages of behavior change, they are considered independent in their capacity to predict and facilitate health behavior modifications.

Despite the MTM model’s robust framework and its success in elucidating health behavior changes, it does not adequately address the development of behavior skills. For instance, the practice of standardized blood glucose monitoring, as well as the capacity for effective communication and interaction with others to secure support and resources, are crucial components of behavior skills in health interventions. Most prior research has concentrated on the motivational aspects of health behaviors, largely overlooking the volitional phases—those that involve the actual enactment and persistence of behavior changes. Given that health behavior is inherently action-oriented and requires sustained effort, identifying factors that bolster the maintenance of health behaviors is imperative. This gap underscores the need for an expanded theoretical framework that not only guides the initiation but also the ongoing maintenance of health behaviors, ensuring more holistic and effective health interventions.

The Health Action Process Approach (HAPA) theory, conceptualized by German psychologist Schwarzer in 1992, is an extension of Bandura’s self-efficacy theory (25). HAPA posits that health behavior change is a sequential process comprising two phases: motivational and volitional. The motivational phase involves the transition from no intention to behave in a certain way to the formation of that intention. This is influenced by an amalgamation of factors including self-efficacy, expected outcomes of behavior, and perceptions of disease risk. The volitional phase, on the other hand, is where intentions are translated into concrete actions. This phase is further categorized into preparation and action/maintenance stages. Challenges such as distractions, external environmental influences, and uncontrollable factors can disrupt this phase. However, individuals endowed with strong self-efficacy are more equipped to navigate these challenges and resume their intended behaviors. Notably, HAPA distinguishes between two types of self-efficacy: action self-efficacy and self-efficacy for sustaining behavior. Action self-efficacy, relevant in the pre-action motivational phase, represents the optimistic belief about achieving success and envisaging the outcomes of various strategies before actual behavior occurs. Conversely, self-efficacy for sustaining behavior, pertinent to the post-action volitional phase, involves confidence in one’s ability to sustain the behavior amidst obstacles. Recent meta-analytical evidence supports the efficacy of HAPA in predicting health behaviors, particularly through mechanisms like planning and self-efficacy structures (26). The theory comprehensively delineates the intricacies of transitioning from motivational genesis to actual behavioral execution, accentuating the pivotal role of planning as a mediator and the critical influence of self-efficacy at each stage (27). While HAPA provides a robust framework for understanding the stages of behavior change, including the formation of behavioral intentions, it does not thoroughly elucidate the direct mechanisms by which these variables induce actual behavior change.

Current scholarly discourse places substantial emphasis on the theoretical foundations necessary for explaining and enhancing health behaviors. Nevertheless, the application of these theories frequently suffers from a narrow focus, missing a holistic perspective (28). The factors influencing health behaviors are myriad and multifaceted, thereby challenging any single theoretical framework to encompass all aspects comprehensively. While various theories are utilized in population health behavior interventions—each with distinctive features—their explanatory and predictive capacities, particularly regarding the initiation and maintenance of behaviors, remain inadequate. Both HAPA and the MTM share structural similarities in their focus on behavior change and maintenance, yet they differ in their treatment of influencing factors and outcome variables. HAPA incorporates mediating variables that bridge the gap from behavioral intention to action—including action planning and coping planning. In contrast, MTM lacks these mediating variables, which HAPA addresses, effectively filling this theoretical void. Nonetheless, despite its strengths, HAPA lacks a detailed account of how exactly these mediating variables facilitate the transition to concrete behavioral change, indicating a potential area for further theoretical refinement (29). The insights from MTM complement this by elucidating the antecedents of intention formation, thereby enhancing the overall understanding of behavior change mechanisms.Therefore, this study integrates and analyzes the factors influencing the transformation and maintenance of health behaviors in diabetic patients based on the MTM-HAPA theoretical framework, with the aim of laying a foundation for improving patients’ health behaviors.

## 3 Materials and methods

### 3.1 Study design and setting

The study was conducted in 45 community health centers in Beijing, China, with the community health centers serving as the units of randomization. Each community health center included 8-10 patients with T2DM. Staff from the community health centers were responsible for recruiting participants. A total of 406 patients with DM voluntarily participated in our programme. Participants were given oral and written information about the purpose, method, and time schedule of the study, and they signed an informed consent form. Then they filled out the questionnaire. A personal code was matched to each participant to ensure anonymity (30). and we used baseline data to construct a structural equation model. To ensure a representative sample, we initially utilized whole cluster sampling to select two districts, Daxing and Shunyi, from among the 18 districts and counties of Beijing. These districts were approached for permission to conduct the study, and both provided affirmative responses. Consequently, a total of 45 communities in Daxing and Shunyi participated —25 from Shunyi and 20 from Daxing.

Recruitment of participants was carried out in these 45 communities. Local enumerators were enlisted for the survey, and prior to data collection, they received extensive training on sampling methods, the operation of research tools, and quality assurance procedures. A preliminary mock survey was conducted to reinforce the training and ensure proficiency in survey implementation. Only enumerators who strictly adhered to the established protocols were allowed to collect data.

For participant outreach, various methods were used including telephone calls, face-to-face meetings, and digital platforms. Potential participants were fully informed about the study’s purpose, methodology, and timeline either verbally or through written materials. Participation required signing an informed consent form, which detailed the study’s procedures and affirmed the participants’ comprehension and consent. Participants were assured of their right to withdraw from the study at any time without penalty, especially if they experienced any discomfort.

To maintain anonymity and protect privacy, the research questionnaire did not include participants’ names but used unique identification numbers instead. During the survey, investigators directly collected critical physical data from participants, such as glycated hemoglobin levels and body mass index. This data collection was complemented by the administration of questionnaires to capture a broad spectrum of health-related information, adhering to stringent confidentiality and ethical guidelines. This comprehensive approach ensured the integrity and reliability of the data collection process.

### 3.2 Participants

The criteria for participant inclusion and exclusion in this study were meticulously defined. Inclusion criteria encompassed: (1) diagnosis of type 2 diabetes mellitus adopts the T2DM diagnostic criteria: (fasting plasma glucose≥7.0 mmol/L or 2 h plasma glucose≥11.1 mmol/L or HbA1c≥6.5%) (31), as evidenced by fasting blood glucose levels exceeding 7.0 mmol/L, OGTT 2-hour blood glucose levels surpassing 11.1 mmol/L, or average glycated hemoglobin (HbA1c) exceeding 6.5%; (2) age between 18 and 75 years; (3) residency within Beijing City, with absences from the domicile not exceeding one month per year; (4) no history of psychotropic medication usage prior to enrollment; (5) proficiency in using a smartphone and familiarity with the basic functions of WeChat; (6) voluntary participation and completion of the informed consent form. Exclusion criteria included: (1) type 1 diabetes mellitus, gestational diabetes, or secondary diabetes; (2) serious cardiovascular, cerebral, renal, ocular, pedal, or neurological complications (e.g., proliferative retinopathy, stage IV nephropathy or higher, creatinine levels above 2 mg/dl, cardiac function class III or lower, sequelae of cerebrovascular accidents, diabetic foot grade I or higher); (3) issues with mobility, confusion, or mental anomalies; (4) current or recent (within the last six months) cancer patients who have undergone radiotherapy or chemotherapy; (5) participation in other similar research projects; (6) unwillingness to cooperate. This targeted approach ensured the recruitment of a specific participant demographic, crucial for the study’s objectives.

The requisite sample size for structural equation modeling is generally recognized to fall within the range of 250 to 500 participants (32). This range is deemed appropriate, particularly in studies involving more than ten variables. In the current investigation, data were collected from 406 participants across 45 communities within the Daxing and Shunyi districts of Beijing. This sample size not only falls well within the recommended range but also ensures the stability of parameter estimation and enhances the statistical power of significance tests conducted within the study. Prior to the survey, preliminary verification was conducted to ascertain that all participants met the specified inclusion criteria. Additionally, data quality was rigorously assessed post-survey to address and rectify any instances of incorrect or incomplete data entries. Consequently, the dataset comprising information from all 406 participants was deemed comprehensive and was included in the final analysis, thereby bolstering the validity of the study’s findings. This study was conducted in strict adherence to the relevant EQUATOR checklist.

### 3.3 Theoretical integration

**Figure 1:**
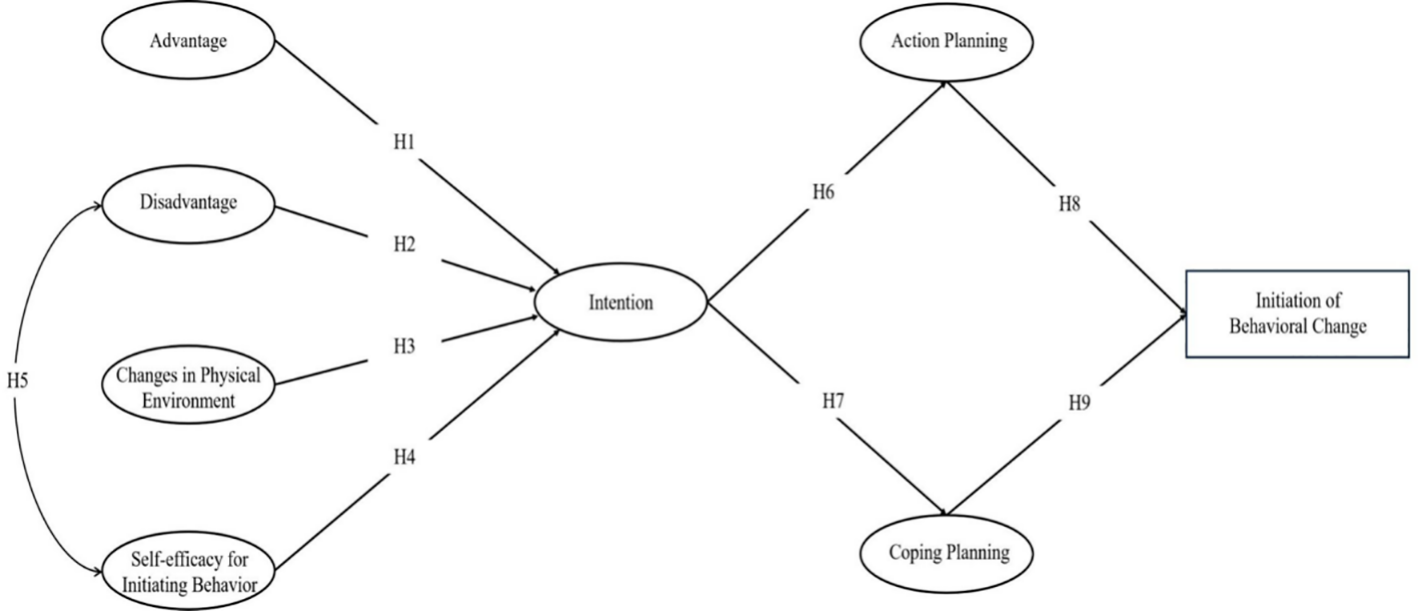
Theoretical integration of MTM-HAPA (Healthy behavior initiation).

**Figure 2:**
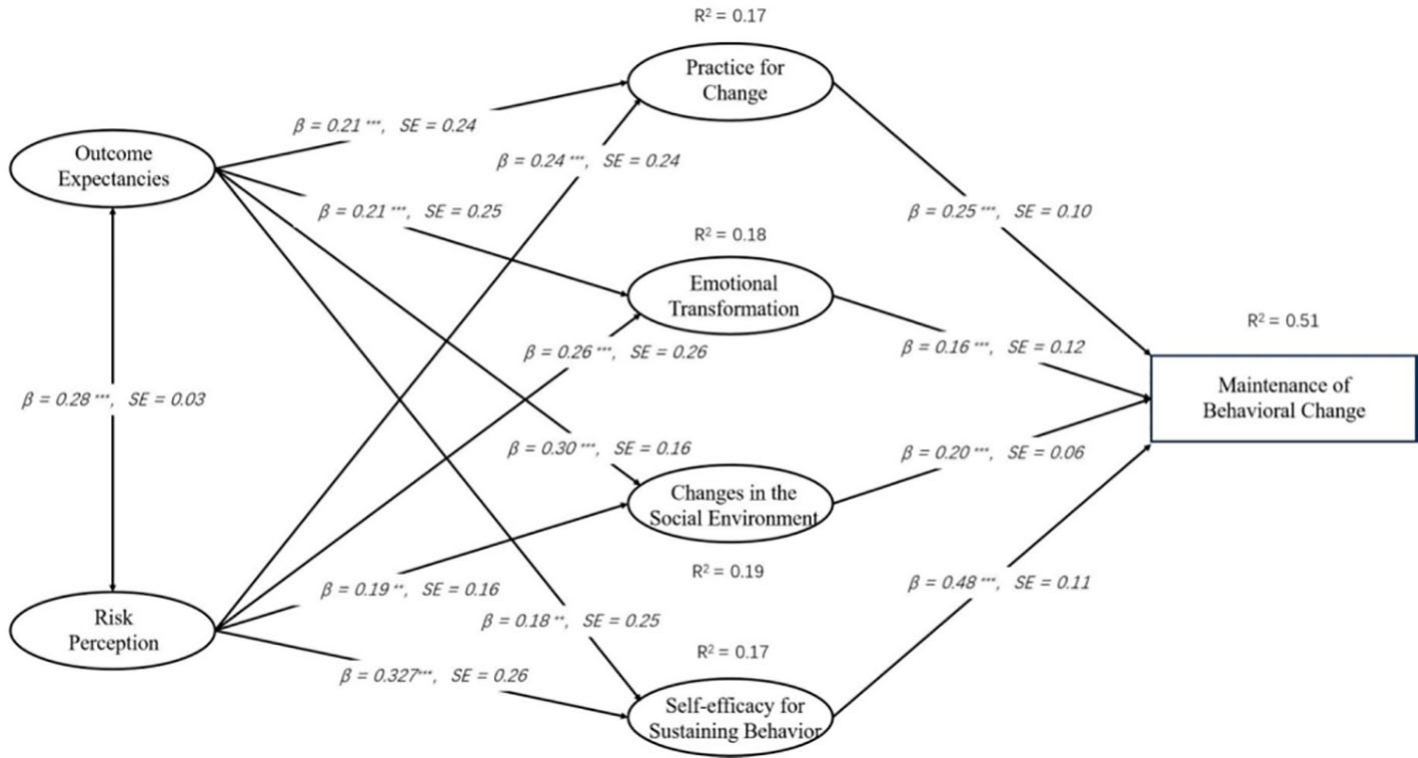
Theoretical integration of MTM-HAPA (Healthy behavior maintenance).

**Figures 1**, **2** depict the theoretical research framework of our study, designed to deepen our comprehension of the factors that prompt behavior change in patients with diabetes. This study intends to establish an integrated MTM-HAPA model and verify the assumptions of the structure and stages of this theoretical model, with the aim of laying the foundation for improving the health behaviors of diabetic patients. Since the MTM and HAPA theories have complementary structures, this study combines the two to explain the health-related behaviors of T2DM patients, forming an MTM-HAPA theoretical framework that includes 15 variables such as a two-way dialogue on the advantages and disadvantages of initiating behavior change, changes in the physical environment, self-efficacy for initiating behavior, behavioral intention, action planning, coping planning, initiation of behavior change, risk perception, outcome expectancies, self-efficacy for sustaining behavior, emotional transformation, practice for change, changes in the social environment, and maintenance of behavioral change. This framework integrates and analyzes the factors affecting the transformation and maintenance of health behaviors in T2DM patients, aiming to lay the foundation for improving patients’ health behaviors. The integration of the two theories focuses on the following three aspects:

1. Integration of Constructs: We incorporated specific constructs from HAPA into the MTM framework that were previously overlooked in the original models. This inclusion aims to enrich our understanding of the dynamics influencing behavior change in patients. Notably, the concept of behavioral intention from HAPA has been added to provide a more nuanced perspective on the factors that shape patients’ behavior changes.
2. Conceptual Distinction of Self-Efficacy and Behavior Confidence: In HAPA, self-efficacy refers to the patients’ current confidence in their ability to change behaviors. In contrast, MTM emphasizes the development of healthy behavior habits over time. MTM’s ‘behavior confidence’ extends this concept by focusing on the certainty of future behaviors, rooted in one’s self-cognition. This shift from immediate to future-oriented confidence is crucial as behavior confidence, compared to self-efficacy, offers a more robust framework for explaining both the change and maintenance of behaviors.
3. Redefinition of Behavioral Phases: HAPA elaborates on the roles of action self-efficacy, coping self-efficacy, and recovery self-efficacy throughout the phases of behavior generation, maintenance, and recovery. Conversely, our study, influenced by the structural framework of MTM, bifurcates behavior into ‘initiation’ and ‘maintenance’ phases. Consequently, we have aligned the study’s framework by incorporating only the self-efficacy related to action and maintenance. This alignment ensures that our model effectively captures the nuances of initiating and sustaining behaviors in diabetic patients.

These adaptations allow for a more precise evaluation of the theoretical constructs and their applicability in predicting and understanding behavior changes in diabetic individuals, enhancing the theoretical robustness and practical relevance of our model.

### 3.4 Measurement

The research incorporated 15 variables derived from the MTM proposed by Sharma (33) and the HAPA (34) theory developed by Schwarzer. To elucidate the factors influencing the initiation and maintenance of behavioral change, these variables were strategically distributed into two models. Model 1 integrated eight of these variables to assess their impact on the Initiation of Behavior Change. Conversely, Model 2 included seven variables to examine their influence on the sustenance and maintenance of behavioral changes. The assessment of all variables was conducted using a five-point Likert scale, facilitating a nuanced understanding of the motivational and process-oriented factors contributing to health-related behavioral modifications. The variables are described in **Tables 1**, **2**.

**Table 1:** The scale description of MTM-HAPA theory in model 1.

	Theoretical dimension	Variable Name	Focused Measurement Content	Theoretical Source	Scoring Method	Scoring Range	Scoring Meaning	Adapted From
Model 1	Participatory Dialogue: Advantage	Advantage	Perceived benefits of engaging in daily health management behaviors across five key areas	MTM	5-point Likert scale	1-5	Higher scores indicate better self-management by the patient.	Sharma et al. (2017) (49)
	Participatory Dialogue: Disadvantage	Disadvantage	Perceived disadvantages of engaging in daily health management behaviors across five key areas	MTM	5-point Likert scale	1-5	Higher scores indicate lower self-management by the patient.	Sharma et al. (2017) (49)
	Behavioral Confidence	Self-efficacy for Initiating Behavior	Participants’ confidence in their ability to initiate health management behaviors under varying conditions of perceived difficult	MTM & HAPA	5-point Likert scale	1-5	Higher scores indicate better self-management by the patient	Schwarzer (2016) (34)
	Changes in Physical Environment	Changes in Physical Environment	Degree of health management conducted by participants across three distinct physical settings	MTM	5-point Likert scale	1-5	Higher scores indicate better self-management by the patient	Sharma (2015) (33)
	Intention	Intention	Extent of participants’ commitment to partake in diverse health-related behaviors over forthcoming periods	HAPA	5-point Likert scale	1-5	Higher scores indicate better self-management by the patient	Schwarzer (2016) (34)
	Action Planning	Action Planning	Thoroughness and clarity of participants’ plans for initiating health management:	HAPA	5-point Likert scale	0-4	Higher scores indicate better self-management by the patient	Schwarzer (2016) (34)
	Coping Planning	Coping Planning	The extent to which participants understand how to develop coping strategies and plans to maintain their health management behaviors when facing specific challenges or obstacles.	HAPA	5-point Likert scale	0-4	Higher scores indicate better self-management by the patient	Schwarzer (2016) (34)
	Initiation of Behavior Change	Initiation of Behavior Change	Likelihood of consistently engaging in health management practices moving forward	MTM	5-point Likert scale	1-5	Higher scores indicate better self-management by the patient	Sharma (2015) (33)

**Table 2:** The scale description of MTM-HAPA theory in model 1.

	Theoretical dimension	Variable Name	Focused Measurement Content	Theoretical Source	Scoring Method	Scoring Range	Scoring Meaning	Adapted From
Model 2	Outcome Expectancies	Outcome Expectancies	Expectations regarding the outcomes of managing their health effectively	HAPA	5-point Likert scale	0-4	Higher scores indicate better self-management by the patient	Schwarzer (2016) (34)
	Risk Perception	Risk Perception	These questions measure the extent to which participants perceive the health risks associated with not managing their health or lacking a good health management plan.	HAPA	5-point Likert scale	0-4	Higher scores indicate better self-management by the patient	Schwarzer (2016) (34)
	Changes in the Social Environment	Changes in the Social Environment	Extent to which participants are confident in receiving support from family members, friends, and medical professionals to manage their health.	MTM	5-point Likert scale	1-5	Higher scores indicate better self-management by the patient	Sharma(2015) (33)
	Emotional Transformation	Emotional Transformation	Extent to which participants are confident in channeling their emotions, encouraging themselves, and overcoming self-doubt to achieve their goal of managing their health on a daily basis.	MTM	5-point Likert scale	1-5	Higher scores indicate better self-management by the patient	Sharma(2015) (33)
	Behavioral Confidence	Self-efficacy for Sustaining Behavior	Participants’ self-efficacy in maintaining their health management under varying conditions of perceived difficult	MTM & HAPA	5-point Likert scale	1-5	Higher scores indicate better self-management by the patient	Schwarzer (2016) (34)
	Practice for Change	Practice for Change	Participants’ confidence in managing their health in different scenarios	MTM	5-point Likert scale	1-5	Higher scores indicate better self-management by the patient	Sharma(2015) (33)
	Sustenance/Long termbehavior change	Maintenance of Behavioral Change	Likelihood of participants initiating daily health management in the following week	MTM	5-point Likert scale	1-5	Higher scores indicate better self-management by the patient	Sharma(2015) (33)

These variables collectively aim to provide a comprehensive understanding of the factors influencing the persistence of health behavior changes, integrating both motivational and practical considerations. **Table 3** shows the specific content of the scale. The scale has good reliability and validity, with a Cronbach’s alpha coefficient of 0.955. The results of the confirmatory factor analysis can be seen in **Table 4**.

**Table 3:** Measurement constructs and items.

Construct	Items
Advantages	If you manage your health every day, you will become healthier.
	If you manage your health every day, your fasting blood sugar will be less than 7 mmol/L.
	If you manage your health every day, your diet will be less oil, less salt, and less sugar.
	If you manage your health every day, add at least 30 minutes of exercise time each day.
	If you manage health on a daily basis, your confidence in the treatment of your illness will increase significantly.
Disadvantages	If you take care of your health every day, it will become very inconvenient
	If you take care of your health every day, you will waste time and energy.
	If you manage your health every day, you will reduce your social activities.
	If you take care of your health every day, you will have an adverse reaction.
	If you manage your health on a daily basis, you will feel anxious, depressed, and stressed.
Self-efficacy for Initiating Behavior	Even if it feels like a hassle, you can trust that you can start managing your health.
	Even if you feel nervous and anxious, you can trust that you can start managing your health.
	Even if you think it’s hard to do, you can start managing your health.
Changes in Physical Environment	How confident are you that you will manage your health regularly every day?
	How confident are you that you will be able to manage your health on a daily basis with the help of an artificial intelligence program
	How confident are you that you will receive knowledge about health management on a daily basis?
Intention	You plan to stick to your health management for the next month.
	You plan to stick to your health management for the next two weeks.
	You plan to start health management as soon as possible.
Action Planning	You have a detailed plan for when to start health management.
	You have a detailed plan for where to start health management.
	You have a detailed plan on how to get started with health management
Coping Planning	When you have the idea of giving up health management, know how to deal with it.
	When your schedule conflicts, know how to manage your time wisely to ensure your health.
	When your body is not feeling well, know how to adjust your state.
Initiation of Behavior Change	What is the likelihood that you will start daily health management in the following week
Outcome Expectancies	If you take care of your health, your body will be better.
	If you manage your health, your blood sugar will be lowered.
	If you manage your health, you will be healthier in your diet and exercise.
Risk Perception	If you don’t manage your health, you’re more likely to develop type 2 diabetes than people of the same age and gender
	If you don’t manage your health, you’re more likely to have an increase in blood sugar
	Without a good health management plan, your chances of developing diabetes complications will increase
Changes in Social Environment	How confident are you that you will have the support of your family members to manage your health?
	How sure are you that you will have the support of your friends to manage your health?
	How confident are you that you will have the support of a medical professional to manage your health?
Emotional Transformation	How confident are you that you’ll be able to channel your emotions to achieve your goal of managing your health every day?
	How sure are you that you can encourage yourself to take care of your health every day?
	How confident are you that you will be able to overcome your self-doubt about managing your health on a daily basis?
Self-efficacy for Sustaining Behavior	How confident are you that you will be able to start managing your health this week?
	How sure you are that you will be able to start health management this week, even if it is inconvenient?
	What is the possibility that you will be taking care of your health every day from now on?
	How confident are you that you will start managing your health this week without any emotion?
	How sure you are that you can start managing your health even if you are out and about?
Practice for change	How confident are you that you can use the app to monitor your daily health management?
	If you encounter obstacles, how confident are you that you will be able to manage your health every day?
	If you get stuck, how sure are you that you’ll be able to adjust your daily health management goals?
Maintenance of Behavioral Change	How confident are you that you will be able to start managing your health this week despite your busy schedule?

**Table 4:** The confirmatory factor analysis results of the MTM-HAPA scale.

Items	χ^2^/df	RMSEA	GFI	TLI	IFI	CFI
Initiation of Behavior Change	2.50	0.04	0.90	0.96	0.96	0.97
Maintenance of Behavioral Change	1.82	0.02	0.93	0.99	0.99	0.99

Beyond the 15 core variables, our questionnaire meticulously gathered fundamental demographic and health-related information about the participants. This encompassed gender, age, glycosylated hemoglobin levels, ethnicity, body mass index (BMI), per capita monthly household income, household registration, and health insurance status. To bolster the questionnaire’s reliability and validity, we engaged experts across multiple disciplines—clinical medicine, public health, psychology, behavioral science, and statistics. These specialists evaluated the questionnaire’s content validity, ensuring that it comprehensively covered the necessary domains pertinent to our study. Through this collaborative, interdisciplinary review, we confirmed the reliability and usability of the questionnaire, laying a solid foundation for the integrity and accuracy of our data collection process. This rigorous approach underscores our commitment to generating robust, actionable insights into the health management behaviors of individuals with diabetes.

### 3.5 Data analyses

The study’s data were digitized and analyzed using IBM SPSS v25.0 software. Initial steps included performing descriptive statistics to summarize the dataset and conducting reliability tests using Cronbach’s alpha coefficient to assess the consistency of the questionnaire items. Correlation analyses were subsequently carried out to determine the relationships among variables, employing the Pearson’s correlation coefficient for this purpose.

For a more in-depth examination of the measurement model’s validity, Confirmatory Factor Analysis (CFA) was conducted using Amos 21.0 software, focusing on evaluating both convergent and discriminant validity. Structural Equation Modeling (SEM) was then utilized to analyze the hypothesized pathways within the model. Commonly accepted criteria for SEM evaluation were applied, including Chi-square/degrees of freedom (χ²/df), Goodness-of-Fit Index (GFI), Comparative Fit Index (CFI), Root Mean Square Error of Approximation (RMSEA), Normal Fit Index (NFI), Tucker-Lewis Index (TLI), and Incremental Fit Index (IFI). These analyses were rigorously performed in accordance with established guidelines.

## 4 Results

### 4.1 Resource identification initiative

**Table 4** delineates the foundational demographics and health metrics of the study cohort. Initial analysis of gender composition reveals a relatively equitable distribution, with males constituting 42.9% and females 57.2%. The participants’ mean age was notably elevated, averaging 56.7 ± 10.9 years. In terms of glycemic management, the HbA1c level stood at 7.4, accompanied by a significant standard deviation of 1.6, indicating variability in diabetes management across the sample. BMI data revealed that 29.6% of participants fell within the normal range, whereas 37.4% and 32.3% were categorized as overweight and obese, respectively, with a minimal underweight fraction of 0.74%. Financially, a majority (55.4%) reported a per capita monthly household income between 3001 to 6000 CNY, and 29.6% earned 3000 CNY or less. Regarding domicile registration, 60.8% were classified as non-agricultural, contrasting with 39.2% as agricultural residents. Health insurance status showed that a predominant 94.6% were beneficiaries of Beijing health insurance, whereas 2.7% were uninsured, and an equal percentage were covered by non-local health insurance policies. This information is exhaustively cataloged in **Table 5**.

**Table 5:** Basic information of the respondents.

Items	Description	Number	Percentage (%)
Gender	Man	174	42.9
	Woman	232	57.1
Ethnicity	Han ethnicity	396	97.5
	Ethnic minority	10	2.5
Body Mass Index(BMI)	Underweight	3	0.7
	Normal	120	29.6
	Overweight	152	37.4
	Obesity	131	32.3
Household monthly income per capita in CNY	≤3000	120	29.6
	3001-6000	225	55.4
	≥6001	61	15.0
Household registration	Non-agricultural	247	60.8
	Agricultural	159	39.2

### 4.2 Reliability analysis

This study utilized the Cronbach’s α coefficient to evaluate the reliability of the questionnaire, aiming to ensure its consistency and stability across various dimensions. The analysis presented in **Table 6** outlines the mean scores and standard deviations (Mean ± SD) for each dimension, in addition to the respective Cronbach’s α coefficients. The observed mean scores and standard deviations reveal a notable dispersion of data points around the mean, underscoring the sample’s diversity and variability. The Cronbach’s α coefficients indicate that the reliability of all questionnaire dimensions is within an acceptable range. Notably, most dimensions demonstrate reliability coefficients surpassing 0.9, reflecting high levels of consistency and stability across the questionnaire’s dimensions. Consequently, the questionnaire deployed in this investigation is confirmed to have high reliability, proficiently capturing the participants’ perspectives and attitudes across a spectrum of behavioral and attitudinal dimensions.

**Table 6:** Mean scores (Mean ± SD) and corresponding Cronbach’s α coefficients.

Questionnaire	Mean ± SD	Cronbach’s α
Advantages	14.5 ± 4.2	0.874
Disadvantages	5.8 ± 6.0	0.952
Self-efficacy for Initiating Behavior	11.6 ± 3.2	0.975
Changes in Physical Environment	6.5 ± 3.6	0.874
Intention	11.6 ± 3.4	0.969
Action Planning	9.3 ± 4.3	0.994
Coping Planning	11.0 ± 3.1	0.922
Outcome Expectancies	12.8 ± 2.1	0.950
Risk Perception	12.4 ± 2.4	0.918
Changes in Social Environment	9.3 ± 3.0	0.942
Emotional Transformation	7.6 ± 3.6	0.980
Self-efficacy for Sustaining Behavior	17.0 ± 6.4	0.986
Practice for change	7.1 ± 3.8	0.953

### 4.3 Pearson’s correlations

In this study, we conducted a correlation analysis to explore the relationships among variables within two distinct models. Model 1 focuses on understanding the factors influencing the Initiation of Behavior Change. In this model, the variables include Advantages, Disadvantages, Self-efficacy for Initiating Behavior, Changes in Physical Environment, Intention, Action Planning, Coping Planning, and the ultimate outcome, Initiation of Behavior Change. In contrast, Model 2 investigates the factors influencing the sustenance of behavioral change. The variables in this model include Outcome Expectancies, Risk Perception, Changes in Physical Environment, Changes in Social Environment, Emotional Transformation, Self-efficacy for Sustaining Behavior, practice for change, and the ultimate outcome, Maintenance of Behavioral Change.

Based on the results in **Table 7**, in Model 1, which focuses on factors influencing the Initiation of Behavior Change, significant associations were observed among the variables. In model 1, which primarily focuses on factors influencing the Initiation of Behavior Change, significant associations were observed among the variables. Firstly, Advantage and Intention are significantly positively correlated (r = 0.426, p < 0.001), this supports hypothesis H1, which predicted that advantage would have a positive impact on behavioral intentions. It indicated that the greater the perceived benefits of daily health management behaviors, the stronger the intention to change behavior. Conversely, Disadvantage and Intention are significantly negatively correlated (r = -0.239, p < 0.001),this supports hypothesis H2, which predicted that disadvantage would have a negative impact on behavioral intentions. It suggested that the fewer the perceived disadvantages of daily health management behaviors, the stronger the intention to change behavior. Changes in Physical Environment and Intention also show a significant positive correlation (r = 0.498, p < 0.001), this supports hypothesis H3, which predicted that Changes in Physical Environment would have a positive impact on behavioral intentions. It implied that improvements in the physical environment can enhance the intention to change behavior. Additionally, Self-efficacy for Initiating Behavior and Intention are significantly positively correlated (r = 0.654, p < 0.001), this supports hypothesis H4, which predicted that Self-efficacy for Initiating Behavior would have a positive impact on behavioral intentions. It indicated that higher self-efficacy for initiating behavior leads to stronger behavioral change intentions. There is also a significant association between Disadvantage and Changes in Physical Environment, which supports hypothesis H5. Intention and Action Planning are significantly positively correlated (r = 0.577, p < 0.001), this supports hypothesis H6, which predicted that intention would have a positive impact on Action Planning. It suggested that stronger behavioral change intentions lead to more detailed action plans. Similarly, Intention and Coping Planning are significantly positively correlated (r = 0.668, p < 0.001), this supports hypothesis H7, which predicted that intention would have a positive impact on Coping Planning. It indicated that stronger behavioral change intentions lead to more proactive coping plans. Further analysis shows that Action Planning and Initiation of Behavior Change are significantly positively correlated (r = 0.492, p < 0.001), this supports hypothesis H9, which predicted that Action Planning would have a positive impact on Initiation of Behavior Change. It suggested that detailed action plans help initiate behavioral change. Coping Planning and Initiation of Behavior Change are also significantly positively correlated (r = 0.602, p < 0.001), this supports hypothesis H9, which predicted that Coping Planning would have a positive impact on Initiation of Behavior Change. It indicated that proactive coping plans aid in the Initiation of Behavior Change.

**Table 7:** Correlation of variables in model 1.

	Advantages	Disadvantages	Changes in Physical Environment	Self-efficacy for Initiating Behavior	Intention	Action Planning	Coping Planning	Initiation of Behavior Change
Advantages								
Disadvantages	-0.083							
Changes in Physical Environment	0.497***	-0.110*						
Self-efficacy for Initiating Behavior	0.459***	-0.266***	0.460***					
Intention	0.426***	-0.239***	0.498***	0.654***				
Action Planning	0.292	0.069	0.485	0.444***	0.577***			
Coping Planning	0.475***	-0.212***	0.510***	0.667***	0.668***	0.563***		
Initiation of Behavior Change	0.394***	-0.247***	0.516***	0.608***	0.715***	0.492***	0.602***	

*P<0.05, ***P<0.001.

Based on the results in **Table 8**, in Model 2, which investigates factors influencing the Sustenance of Behavioral Change, significant correlations were also found among the variables. Firstly, Outcome Expectancies and Practice for Change show a significant positive correlation (r = 0.324, p < 0.001), this supports hypothesis H10, which predicted that Outcome Expectancies would have a positive impact on Practice for Change. It indicated that positive outcome expectancies contribute to enhanced practice for change. Similarly, Outcome Expectancies are positively correlated with Emotional Transformation (r = 0.338, p < 0.001), Changes in the Social Environment (r = 0.384, p < 0.001), and Self-efficacy for Sustaining Behavior (r = 0.316, p < 0.001), these support hypothesis from H11 to H13, which predicted that Outcome Expectancies would have positive impacts on Emotional Transformation, Changes in the Social Environment, and Self-efficacy for Sustaining Behavior. It suggested that they also enhance emotional transformation, social environment changes, and self-efficacy. Risk Perception is positively correlated with Practice for Change (r = 0.327, p < 0.001), Emotional Transformation (r = 0.344, p < 0.001), and Changes in the Social Environment (r = 0.341, p < 0.001), these support hypothesis from H14 to H16, which predicted that Risk Perception would have positive impacts on Practice for Change, Emotional Transformation, and Changes in the Social Environment. It indicated that higher risk perception facilitates practice for change, emotional transformation, and changes in social support. Additionally, Risk Perception and Self-efficacy for Sustaining Behavior (r = 0.342, p < 0.001) are positively correlated, this supports hypothesis H17, which predicted that Risk Perception would have a positive impact on Self-efficacy for Sustaining Behavior. It suggested that higher risk perception also enhances self-efficacy. Further analysis shows significant positive correlations between Practice for Change and the Maintenance of Behavioral Change (r = 0.727, p < 0.001), this supports hypothesis H18, which predicted that Practice for Change would have a positive impact on the Maintenance of Behavioral Change. It indicated that active practice for change aids in the maintenance of behavioral change. Similarly, Emotional Transformation (r = 0.754, p < 0.001) and Changes in the Social Environment (r = 0.565, p < 0.001) are positively correlated with the maintenance of behavioral change, these support hypothesis H19 and H20, which predicted that Emotional Transformation and Changes in the Social Environment would have positive impacts on the Maintenance of Behavioral Change. It highlighted the importance of emotional and social factors. Finally, Self-efficacy for Sustaining Behavior has a strong positive correlation with the maintenance of behavioral change (r = 0.776, p < 0.001), this supports hypothesis H21, which predicted that Self-efficacy for Sustaining Behavior would have a positive impact on the Maintenance of Behavioral Change. It indicated that higher self-efficacy leads to more successful maintenance.

**Table 8:** Correlation of variables in model 2.

	Outcom Expectancies	Risk Perception	Practice for change	Emotional transformation	Changes in the Social Environment	Self-efficacy for Sustaining Behavior	Maintenance of Behavioral Change
Outcom Expectancies							
Risk Perception	0.563***						
Practice for change	0.324***	0.327***					
Emotional Transformation	0.338***	0.344***	0.824***				
Changes in the Social Environment	0.384***	0.341***	0.507***	0.581***			
Self-efficacy for Sustaining Behavior	0.316***	0.342***	0.835***	0.888***	0.553***		
Maintenance of Behavioral Change	0.312***	0.343***	0.727***	0.754***	0.565***	0.776***	

***P<0.001.

To address the challenge posed by the violation of the multivariate normality assumption in the dataset, the Bollen-Stine Bootstrap method was employed as a corrective measure prior to conducting SEM analysis. This approach involves resampling the data (bootstrap) to generate new sample distributions, which are then used to estimate the standard errors and confidence intervals of the model parameters. By leveraging a bootstrap resampling technique that does not depend on the distribution of the data, it enables the accurate estimation of parameter variances and covariances, thereby enhancing the robustness and reliability of the path analysis results.

The efficacy of the SEM was evaluated through a comprehensive examination of the model’s fit indexes and the variance-explained estimates. This rigorous assessment ensures that the SEM not only accurately reflects the relationships among the variables but also provides meaningful insights into the mechanisms underlying the initiation and sustenance of behavioral changes.

The fit indices for Model 1 underscored a robust support for the hypothesized model in this research. The ratios and indices fell within ideal ranges: χ²/df ratio was significantly below the threshold of 3, standing at 1.37, indicating a good fit. The GFI was 0.97, exceeding the recommended level of 0.9, alongside the TLI, Incremental Fit Index (IFI), and CFI each displaying a value of 0.99, well above the 0.9 mark. The RMSEA was notably low at 0.03, further validating the model’s fit (refer to **Table 9** for detailed metrics) (35).

**Table 9:** The quality of the SEM 1.

χ^2^/df	RMSEA	GFI	TLI	IFI	CFI
1.37	0.03	0.97	0.99	0.99	0.99

**Figure 3:**
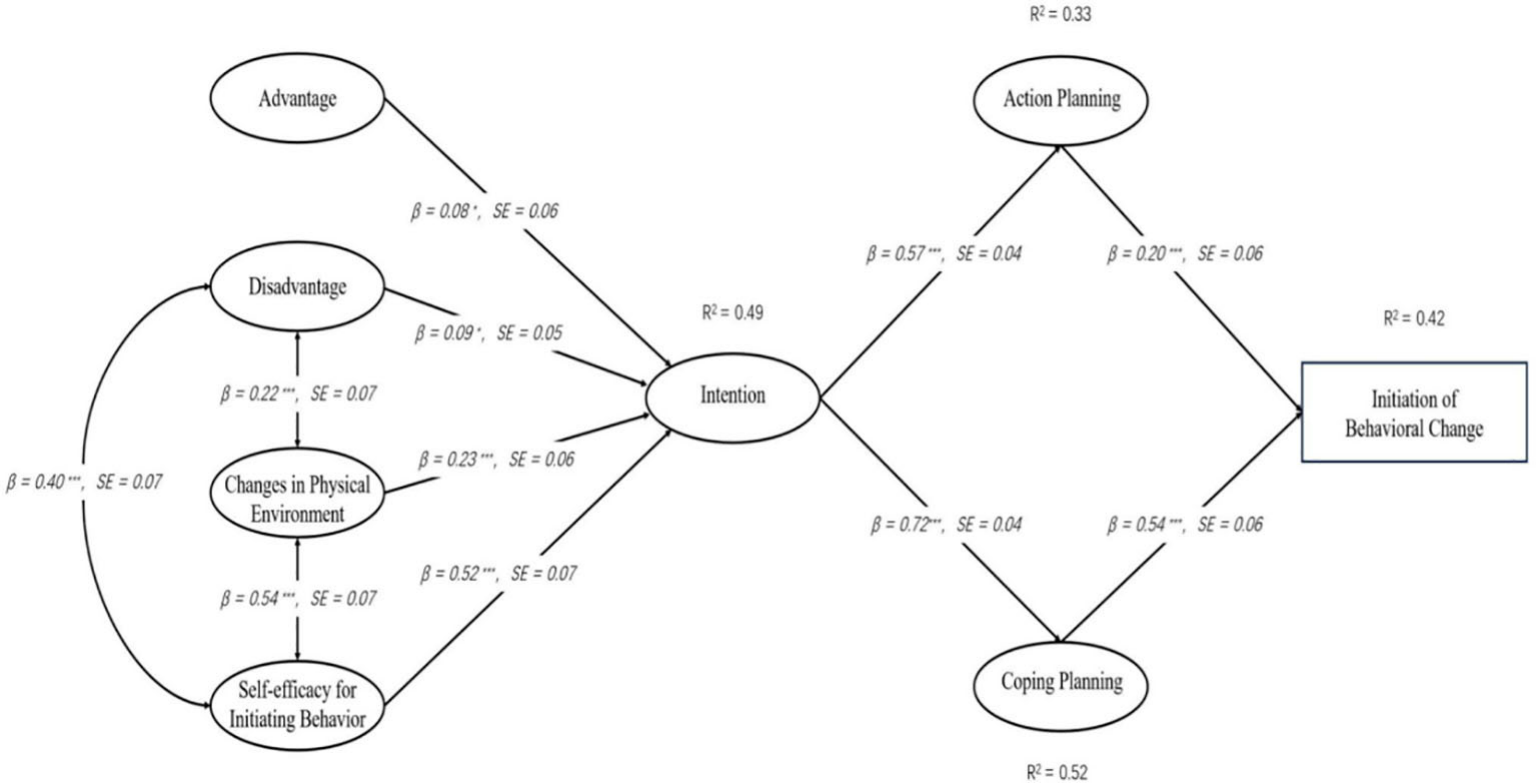
MTM-HAPA path models with standardized parameter estimates to predict the initiation of healthy behavior.

The **Figure 3** presents the results of the path analysis exploring the relationships among variables in model 1. The data indicates significant pathways between the variables. Disadvantages negatively influence Intention (β= -0.086, p < 0.05), while Advantages (β= 0.082, p < 0.05), Self-efficacy for Initiating Behavior (β= 0.520, p < 0.001) and Changes in Physical Environment (β= 0.232, p < 0.001) positively impact Intention. Of these, Self-efficacy for Initiating Behavior had the strongest positive effect on Intention. Intention, in turn, positively influences both Action Planning (β= 0.573, p < 0.001) and Coping Planning (β= 0.724, p < 0.001). Furthermore, Action Planning (β= 0.195, p < 0.001) and Coping Planning (β= 0.539, p < 0.001) significantly predict Initiation of Behavior Change, indicating their crucial roles in facilitating behavior change. Notably, the data showed that intentions had a greater effect on Coping Planning than on Action Planning, and Coping Planning had a greater effect on Initiation of Behavior Change than action planning.Therefore, the assumptions of H1 through H9 are valid.

### 4.4 Factors influencing the maintenance of behavioral change

In preparing to analyze Model 2, the Bollen-Stine Bootstrap method was once again applied to account for data corrections. The fitness indicators for Model 2 were within the acceptable range, reinforcing the model’s credibility. Specifically, the χ²/df ratio was 1.69, GFI reached 0.98, and TLI, IFI, and CFI values were at 0.99, all surpassing the 0.9 benchmark. The RMSEA stood at a commendable 0.04 (details available in **Table 10**).

**Table 10:** The quality of the SEM 2.

χ^2^/df	RMSEA	GFI	TLI	IFI	CFI
1.69	0.04	0.98	0.99	0.99	0.99

**Figure 4:**
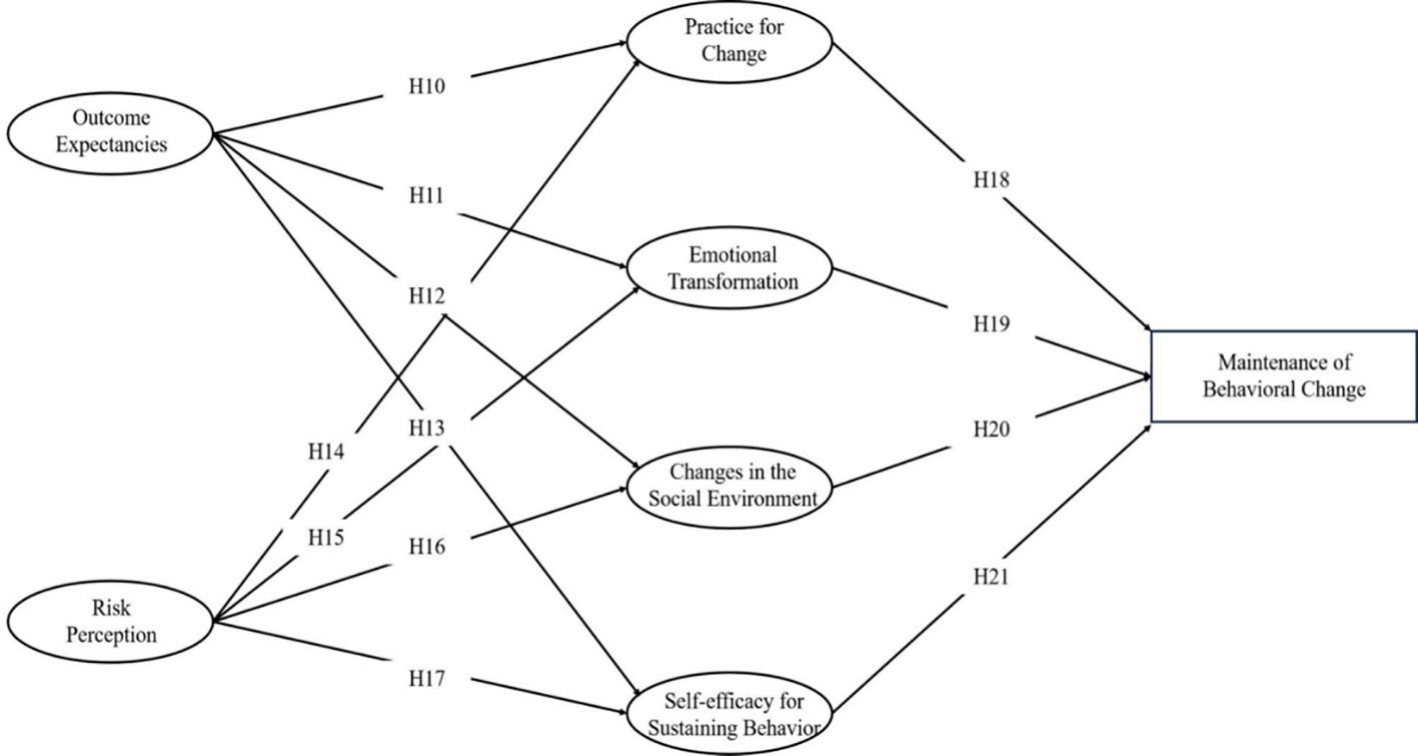
MTM-HAPA path models with standardized parameter estimates to predict the maintenance of healthy behavior.

Path analysis of Model 2 revealed significant relationships among variables, illustrating the dynamic interplay influencing the sustenance of behavioral change. Outcome Expectancies were a positive predictor of Practice for change (β= 0.212, p < 0.001) and also significantly associated with Emotional Transformation (β= 0.214, p < 0.001) and Changes in Social Environment (β= 0.297, p < 0.001). Risk Perception was another significant predictor of Practice for change (β= 0.241, p < 0.001), Emotional Transformation (Estimate = 0.260, p < 0.001), and Changes in Social Environment (Estimate = 0.188, p = 0.002). Notably, both Outcome Expectancies (β= 0.183, p = 0.003) and Risk Perception (β= 0.270, p < 0.001) positively influenced Self-efficacy for Sustaining Behavior. Furthermore, Practice for change was a significant predictor of Maintenance of Behavioral Change (β= 0.250, p < 0.001), with Emotional Transformation (β= 0.160, p < 0.001), Changes in Social Environment (β= 0.200, p < 0.001), and particularly Self-efficacy for Sustaining Behavior (β= 0.484, p < 0.001) playing pivotal roles in this relationship (refer to **Figure 4** for an illustrative overview). Therefore, the assumptions of H10 through H21 are valid.

## 5 Discussion

This study aimed to develop and test two structural equation models, one elucidating how diabetes patients’ perceptions of the pros and cons of health behavior change, changes in physical environment, and self-efficacy for initiating behavior influence their behavioral intentions, as well as the relationships between diabetic patients’ behavioral intentions, action plans, coping plans, and Initiation of Behavior Change. The other model clarified the relationships between emotional transformation, practice for change, changes in social environment, self-efficacy for sustaining behavior, and the impact of their risk perceptions and experiences on these relationships. This process is based on the fourth generation of the MTM and the HAPA model for health behavior change in diabetic patients.

The results are encouraging. In the behavior initiation model, the findings are consistent with predictions, indicating that the pros and cons of behavior change, changes in physical environment, and self-efficacy for initiating behavior are statistically significant predictors of intention. Self-efficacy for initiating behavior was found to be the strongest predictor of intention, underscoring its crucial role. Coping and action plans are statistically significant predictors of the onset of behavior change. Specifically, coping plans play a significant role in predicting behavior change, similar to findings in other studies that emphasize the importance of self-efficacy and planning in health behavior change processes (36). This study supports the notion that behavioral intentions influence coping plans and action plans, which in turn affect actual behavior change. Patients with higher intentions are more likely to develop effective coping and action plans, ultimately leading to successful initiation of behavior change.

In the behavior maintenance model, self-efficacy for sustaining behavior has the strongest predictive power in maintaining behavior among patients, consistent with previous studies. A meta-analysis on HAPA in health behavior found that action self-efficacy and risk perception play significant roles in predicting intentions (26). This highlights the importance of maintaining self-efficacy throughout the behavior change process. The study also reveals that patients’ perceptions of risk and their past experiences significantly impact their ability to sustain behavior change. Patients who perceive higher risks associated with not changing their behavior are more likely to sustain their new behaviors. Additionally, the role of social support in sustaining behavior change is underscored, as changes in the social environment, including support from family and friends, can significantly influence the maintenance of new health behaviors.

This study reveals how patients form or adjust their behavioral intentions based on their assessment of the benefits and harms of health behaviors, changes in the surrounding physical environment, and self-efficacy for initiating behavior. Through in-depth analysis, participants such as patients and health educators can effectively communicate about the pros and cons of specific health behaviors through interactive forms such as group discussions (33), thereby reinforcing the positive aspects of behavior change. This approach encourages participants to realize that the benefits of behavior change far outweigh its potential adverse effects, thereby motivating their willingness to change their behavior. Interactive communication allows for the sharing of personal experiences and success stories, which can further enhance motivation and commitment to behavior change. Health educators can use these discussions to address misconceptions and provide accurate information about the benefits of health behaviors.

Different from the traditional understanding of self-efficacy, under the framework of the MTM, self-efficacy is defined as an individual’s certainty and confidence in his or her self-perception of future behavior change (37, 38). The results of this study confirm the important role of self-efficacy for initiating behavior in predicting behavioral intention, indicating that an individual’s confidence in his or her ability to change behavior is a key factor in forming behavioral intention. This finding underscores the need for interventions that build and support self-efficacy in patients. Techniques such as goal setting, self-monitoring, and feedback can be effective in enhancing self-efficacy. Encouraging patients to reflect on their past successes and to visualize future achievements can also strengthen their confidence in their ability to initiate and sustain behavior changes.

In addition, the findings of this study are also consistent with the theory of reasoned action, which holds that individuals’ beliefs and subjective norms form specific attitudes (i.e., behavioral tendencies), which in turn influence their behavioral intentions (39). This theory suggests that changing patients’ beliefs and norms about health behaviors can lead to changes in their intentions and, subsequently, their behaviors. Furthermore, this study emphasizes the impact of the physical environment on behavioral intentions, pointing out that environmental changes create conditions for behavior change by changing the availability, accessibility, convenience, and readiness of resources. This suggests that by optimizing the physical environment, patients can increase their chances of adopting healthy behaviors, thereby helping to improve their behavioral intentions. For example, making healthy food options more readily available and accessible, creating safe and inviting spaces for physical activity, and providing resources and support for behavior change can all contribute to a more conducive environment for healthy behaviors. The study suggests that comprehensive health promotion strategies should consider both individual psychological factors and physical environmental factors to promote effective behavior change (40). This multifaceted approach can enhance the overall impact of interventions aimed at improving health behaviors among diabetic patients.

Although having good health behavior intentions is a positive first step, intentions do not always translate directly into actual health behaviors. Research by German scholars supports this view (41), emphasizing that the key step in converting intentions into actual actions is through the development of a series of detailed behavioral plans. The early stages of the Health Action Process Approach (HAPA) placed special emphasis on the role of planning in bridging intentions into action (42). This theoretical framework posits that behavioral intentions motivate individuals to set specific health goals and develop action plans to change their behavior. An action plan details the specific steps needed to achieve a goal, including where, when, and how to implement them. These plans help individuals prefigure specific steps they will take to change their health behaviors. Furthermore, by specifying the context and conditions under which the actions will be taken, action plans can make the intended behaviors more concrete and easier to execute.

Closely linked to action plans are coping plans, which are designed to anticipate and plan how to respond to potential obstacles and challenges that may prevent the implementation of the action plan. The purpose of the coping plan is to ensure that the action plan can be executed smoothly without interference from external factors. For example, in patients with diabetes, if the patient plans to exercise moderately after dinner to lower blood sugar levels, adverse weather conditions may interfere with their plans. However, if patients make a plan ahead of time, such as exercising on an indoor treadmill, watching a fitness video, or doing 10 minutes of resistance band or weight training, their risk of deviating from their original plan is greatly reduced. This suggests that using action plans in conjunction with coping plans can be more effective in promoting health behavior change than relying on action plans alone. Therefore, clinicians and health professionals should encourage patients to develop both action and coping plans to enhance the likelihood of carrying out designated health behavior goals. Through this approach, health behavioral intentions can be more effectively translated into actions, leading to long-term health improvements (43).

In the structure of maintaining health behavior change, patients’ outcome expectations and perceived risks can predict the sustainability of their behaviors through changes in their practice, emotions, social environment, and self-efficacy. Within the framework of the HAPA theory (42), outcome expectations and risk perceptions are considered crucial pathways for behavior maintenance. Patients with high perceived risks or who have high outcome expectations are motivated to adopt more protective behaviors. For instance, diabetic patients with high perceived risks or who have high outcome expectations are more inclined to actively screen for retinopathy to prevent complications. This proactive approach is driven by the understanding that early detection and intervention can significantly mitigate the risks associated with diabetes-related complications.

Moreover, when patients perceive risks and anticipate potential outcomes, they might seek advice, comfort, listening, understanding, or practical help from various sources, including family and friends, to gain social support. This social support network plays a pivotal role in reinforcing the patients’ commitment to their health behavior changes. Generally, individuals who perceive a high level of social support tend to be healthier and better at coping with stress (44), making this support crucial for patients to maintain their efforts in adopting healthy behaviors, especially when facing challenges or setbacks. By receiving encouragement, understanding, and practical help from their social networks, patients are more likely to persist in their health behavior changes, thereby improving health outcomes. Social support can also provide emotional reassurance, which is vital for maintaining motivation and overcoming the psychological barriers that often accompany chronic health conditions like diabetes.

Consistent with previous research (45), our findings indicate that higher levels of risk perception are associated with higher levels of behavior change. In other words, diabetic patients who perceive high risks or have high outcome expectations are more likely to take proactive measures to control the disease, which is beneficial for maintaining long-term behavior changes (46). Outcome expectation, which is the anticipated result a patient expects from undergoing treatment, can often motivate an individual to cease a harmful behavior. For example, expecting better glucose management after controlling their sugar intake can drive patients to adhere to dietary recommendations. However, if expectations are too high or unrealistic, such as expecting rapid recovery from high blood sugar levels, they may lead to negative progress, resulting in feelings of discouragement and failure. It is therefore important for health professionals to help patients set realistic and achievable goals, ensuring that their outcome expectations are aligned with their capabilities and the realities of their condition.

Additionally, our findings indicate a correlation between patient self-efficacy, risk perception, and outcome expectations, consistent with previous research (47). Individuals with high perceived risks tend to have higher self-efficacy. Patients with high risk perception and outcome expectations are generally better equipped to handle emergencies, which often results in higher self-efficacy and, consequently, better maintenance of behavior change (48). This enhanced self-efficacy empowers patients to confidently engage in and sustain health-promoting behaviors, thereby contributing to improved long-term health outcomes. Enhanced self-efficacy also facilitates resilience, enabling patients to recover more quickly from setbacks and maintain their commitment to health behavior changes over time. Overall, understanding and leveraging these psychological constructs can significantly enhance the design and implementation of interventions aimed at promoting sustainable health behavior changes among patients. By integrating strategies that build self-efficacy, manage outcome expectations, and provide robust social support, health professionals can create more effective programs that encourage lasting behavior change in diabetic patients.

## 6 Highlights and limitations

To the best of our knowledge, this study represents the first effort to apply the MTM and the HAPA frameworks to delineate the dynamics of health behavior changes. In our quest for precise insights, we meticulously collected extensive data across a broad spectrum of variables and fine-tuned our analytical methods to yield robust findings. However, our study is not without its limitations. First and foremost, the cross-sectional design we adopted, while efficient for simultaneous data collection on various factors, inherently limits our ability to infer causality between independent and dependent variables. Future research could benefit from employing experimental or longitudinal study designs that manipulate variables over time, thereby providing stronger evidence for the effectiveness of the MTM and HAPA models in influencing health behavior changes. Furthermore, the study was conducted only in Beijing with a limited sample size, which may result in relatively low heterogeneity of the study population.The sociocultural and environmental factors unique to Beijing may not necessarily reflect those of other regions, thus limiting the generalizability of our results to different contexts within China or internationally. By acknowledging these limitations, we aim to pave the way for future research to build upon our findings, further refine these theoretical models, and expand the understanding of health behavior changes across diverse populations and settings.

## Data Availability

The raw data supporting the conclusions of this article will be made available by the authors, without undue reservation.

## References

[1] Ahmad E Lim S Lamptey R Webb DR Davies MJ. Type 2 diabetes. Lancet. (2022) 400:1803–20. doi: 10.1016/S0140-6736(22)01655-5 36332637

[2] Sun H Saeedi P Karuranga S Pinkepank M Ogurtsova K Duncan BB. IDF Diabetes Atlas: Global, regional and country-level diabetes prevalence estimates for 2021 and projections for 2045. Diabetes Res Clin Pract. (2022) 183:109119. doi: 10.1016/j.diabres.2021.109119 34879977 PMC11057359

[3] Magliano DJ Boyko EJ IDF Diabetes Atlas 10th edition scientific committee. IDF Diabetes Atlas. 10th edition. Brussels: International Diabetes Federation (2021). 35914061

[4] van Ommen B Wopereis S van Empelen P van Keulen HM Otten W Kasteleyn M. From diabetes care to diabetes cure-the integration of systems biology, eHealth, and behavioral change. Front Endocrinol (Lausanne). (2017) 8:381. doi: 10.3389/fendo.2017.00381 29403436 PMC5786854

[5] Ali MK Pearson-Stuttard J Selvin E Gregg EW. Interpreting global trends in type 2 diabetes complications and mortality. Diabetologia. (2022) 65:3–13. doi: 10.1007/s00125-021-05585-2 34837505 PMC8660730

[6] Martino G Bellone F Langher V Caputo A Catalano A Quattropani MC. Alexithymia and psychological distress affect perceived quality of life in patients with type 2 diabetes mellitus. Mediterr J Clin Psychol. (2019) 7. doi: 10.6092/2282-1619/2019.7.2328

[7] Sridhar GR. On psychology and psychiatry in diabetes. Indian J Endocrinol Metab. (2020) 24:387. doi: 10.4103/ijem.IJEM_188_20 33489842 PMC7810053

[8] Ademoyegun AB Afolabi OE Aghedo IA Adelowokan OI Mbada CE Awotidebe TO. The mediating role of sedentary behaviour in the relationship between social support and depression among individuals with diabetes. Mediterr J Clin Psychol. (2022) 10. doi: 10.13129/2282-1619/MJCP-3420

[9] Mann EA Binder AT Young HN Moreno MA Cox ED. Factors associated with health psychology use in pediatric type 1 diabetes. Diabetes Res Clin Practice. (2020) 161:108071. doi: 10.1016/j.diabres.2020.108071 PMC7078029 32057961

[10] Schmitt A Bendig E Baumeister H Hermanns N Kulzer B. Associations of depression and diabetes distress with self-management behavior and glycemic control. Health Psychol. (2021) 40:113–24. doi: 10.1037/hea0001037 33252963

[11] Pușcașu A Bolocan A Păduraru DN Salmen T Bica C Andronic O. The implications of chronic psychological stress in the development of diabetes mellitus type 2. Mediterr J Clin Psychol. (2022) 10. doi: 10.13129/2282-1619/mjcp-3544

[12] Snoek FJ Anarte-Ortiz MT Anderbro T Cyranka K Hendrieckx C Hermanns N. Roles and competencies of the clinical psychologist in adult diabetes care—A consensus report. Diabetic Med. (2024) 41:e15312. doi: 10.1111/dme.15312 38385984

[13] Caputo A Vicario CM Cazzato V Martino G. Editorial: psychological factors as determinants of medical conditions, volume II. Front Psychol. (2022) 13. doi: 10.3389/fpsyg.2022.865235 PMC8977586 35386893

[14] Marchini F Langher V Napoli A Balonan JT Fedele F Martino G. Unconscious loss processing in diabetes: associations with medication adherence and quality of care. Psychoanalytic Psychotherapy. (2021) 35:5–23. doi: 10.1080/02668734.2021.1922492

[15] Aghili SM Farhang A. Investigating the effectiveness of dialectical behavior therapy on resilience, psychological flexibility, and glycemic control of diabetic patients. Internal Med Today. (2022) 28:514–29. doi: 10.32598/hms.28.4.3866.2

[16] Liu Y Ning X Zhang L Long J Liang R Peng S. Prevalence of long-term complications in inpatients with diabetes mellitus in China: a nationwide tertiary hospital-based study. BMJ Open Diabetes Res Care. (2022) 10:e002720. doi: 10.1136/bmjdrc-2021-002720 PMC9096476 35545316

[17] Middleton KR Anton SD Perri MG. Long-term adherence to health behavior change. Am J Lifestyle Med. (2013) 7:395–404. doi: 10.1177/1559827613488867 27547170 PMC4988401

[18] Wu Y Zhang J Ge Pu Duan T Zhou J Wu Y. Application of chatbots in self-management of diabetes patients: A systematic review and meta-analysis. J Med Internet Res. (2024). 10.2196/60380 PMC11653048 39626235

[19] Sun X Jiang Y Wang J Fan S Fu X An Z. Effects of a mobile health intervention based on a multitheoretical model of health behavior change on anxiety and depression, fear of cancer progression, and quality of life in patients with differentiated thyroid cancer: A randomized controlled trial. BMC Med. (2024) 22:466. doi: 10.1186/s12916-024-03652-0 39407174 PMC11475815

[20] Short SE Mollborn S. Social determinants and health behaviors: conceptual frames and empirical advances. Curr Opin Psychol. (2015) 5:78–84. doi: 10.1016/j.copsyc.2015.05.002 26213711 PMC4511598

[21] Secinti E Wu W Kent EE Demark-Wahnefried W Lewson AB Mosher CE. Examining health behaviors of chronic disease caregivers in the U.S. Am J Prev Med. (2022) 62:e145–58. doi: 10.1016/j.amepre.2021.07.004 34579984

[22] Sharma M Asare M Lakhan R Kanekar A Nahar VK Moonie S. Can the multi-theory model (MTM) of health behavior change explain the intent for people to practice meditation? J Evid Based Integr Med. (2021) 26:2515690X211064582. doi: 10.1177/2515690X211064582 PMC8671666 34898284

[23] Huang M Wang W Sun X Wang Y-J Min H Sun X. A review of multi-theoretical models of health behavior change. Modern Prev Med. (2022) 49:3396–402. doi: 10.20043/j.cnki.MPM.202202113

[24] Sharma M Catalano HP Nahar VK Lingam V Johnson P Ford MA. Using multi-theory model to predict initiation and sustenance of small portion size consumption among college students. Health Promot Perspect. (2016) 6:137–44. doi: 10.15171/hpp.2016.22 PMC5002880 27579257

[25] Wu W Hu L Chen Y Cao F Ding S Wu T. Effectiveness of an online application of the health action process approach (HAPA) theory on oral hygiene intervention in young adults with fixed orthodontic appliances: a randomized controlled trial. BMC Oral Health. (2022) 22:192. doi: 10.1186/s12903-022-02219-w 35590291 PMC9118762

[26] Zhang C-Q Zhang R Schwarzer R Hagger MS. A meta-analysis of the health action process approach. Health Psychol. (2019) 38:623–37. doi: 10.1037/hea0000728 30973747

[27] Lu F Zhang L Xu X Zhai W Ji S Lin L. Effects of multi-dimensional nursing based on HAPA theory on self-care ability and cardiac function in patients with coronary heart failure and heart failure. Altern Ther Health Med. (2023) 29:601–7. 37678861

[28] Rajabi Majd N Broström A Ulander M Lin C-Y Griffiths MD Imani V. Efficacy of a theory-based cognitive behavioral technique app-based intervention for patients with insomnia: randomized controlled trial. J Med Internet Res. (2020) 22:e15841. doi: 10.2196/15841 32234700 PMC7160702

[29] Malik K Amir N Kusumawardhani A Lukman PR Karnovinanda R Melisa L. Health action process approach (HAPA) as a framework to understand compliance issues with health protocols among people undergoing isolation at emergency hospital for COVID-19 Wisma Atlet Kemayoran and RSCM Kiara ultimate Jakarta Indonesia. Front Psychiatry. (2022) 13:871448. doi: 10.3389/fpsyt.2022.871448 35722553 PMC9199900

[30] Wu Y Min H Li M Shi Y Ma A Han Y. Effect of Artificial Intelligence-based Health Education Accurately Linking System (AI-HEALS) for Type 2 diabetes self-management: protocol for a mixed-methods study. BMC Public Health. (2023) 23:1325. doi: 10.1186/s12889-023-16066-z 37434126 PMC10334542

[31] Society CD. Guideline for the prevention and treatment of type 2 diabetes mellitus in China (2020 edition). Chin J Diabetes Mellitus. (2021) 13:315–40. doi: 10.3760/cma.j.cn112138-20211027-00751

[32] Schumacker R Lomax R. A beginner’s guide to structural equation modeling. (1996).

[33] Kapukotuwa S. Nerida T. Batra K. Sharma M. (2024). Interpreting health behaviors using a multitheoretical model (MTM) of health behavior change: a systematic review. Health Promotion Perspectives 14(2):121–135. doi: 10.34172/hpp.42887 39291044 PMC11403345

[34] Schwarzer R. Health action process approach (HAPA) as a theoretical framework to understand behavior change. Actualidades en Psicología. (2016) 30:119–30. doi: 10.15517/ap.v30i121.23458

[35] Hasman A. An introduction to structural equation modeling. Stud Health Technol Inform. (2015) 213:3–6. 26152937

[36] Pilch I Wardawy P Probierz E. The predictors of adaptive and maladaptive coping behavior during the COVID-19 pandemic: The Protection Motivation Theory and the Big Five personality traits. PloS One. (2021) 16:e0258606. doi: 10.1371/journal.pone.0258606 34665837 PMC8525766

[37] Ajzen I. The theory of planned behavior. Organizational Behav Hum Decision Processes. (1991) 50:179–211. doi: 10.1016/0749-5978(91)90020-T

[38] Bandura A. Social foundations of thought and action: A social cognitive theory. Englewood Cliffs, NJ, US: Prentice-Hall, Inc (1986).

[39] LaCaille L. Theory of reasoned action. In: Gellman MD Turner JR, editors. Encyclopedia of Behavioral Medicine. Springer, New York, NY (2013). p. 1964–7.

[40] Sniehotta FF Scholz U Schwarzer R. Bridging the intention–behaviour gap: Planning, self-efficacy, and action control in the adoption and maintenance of physical exercise. Psychol Health. (2005) 20:143–60. doi: 10.1080/08870440512331317670

[41] Wieber F Thürmer JL Gollwitzer PM. Promoting the translation of intentions into action by implementation intentions: behavioral effects and physiological correlates. Front Hum Neurosci. (2015) 9:395. doi: 10.3389/fnhum.2015.00395 26236214 PMC4500900

[42] Schmidt LI Schlomann A Gerhardy T Wahl HW. “Aging Means to Me… That I Feel Lonely More Often”? An Experimental Study on the Effects of Age Simulation Regarding Views on Aging. Frontiers in psychology (2022) 13, 806233. doi: 10.3389/fpsyg.2022.806233 35295394 PMC8918585

[43] Kwasnicka D Presseau J White M Sniehotta FF. Does planning how to cope with anticipated barriers facilitate health-related behaviour change? A systematic review. Health Psychol Review. (2013) 7:129–45. doi: 10.1080/17437199.2013.766832

[44] Zhao G Xie F Li S Ding Y Li X Liu H. The relationship between perceived social support with anxiety, depression, and insomnia among Chinese college students during the COVID-19 pandemic: The mediating role of self-control. Front Psychiatry. (2022) 13:994376. doi: 10.3389/fpsyt.2022.994376 36276317 PMC9582516

[45] Qin H Sanders C Prasetyo Y Syukron M Prentice E. Exploring the dynamic relationships between risk perception and behavior in response to the Coronavirus Disease 2019 (COVID-19) outbreak. Soc Sci Med. (2021) 285:114267. doi: 10.1016/j.socscimed.2021.114267 34388619 PMC8305223

[46] Lu P Kong D Shelley M. Risk perception, preventive behavior, and medical care avoidance among American older adults during the COVID-19 pandemic. J Aging Health. (2021) 33:577–84. doi: 10.1177/08982643211002084 33779385

[47] Pförtner T-K Dohle S Hower KI. Trends in educational disparities in preventive behaviours, risk perception, perceived effectiveness and trust in the first year of the COVID-19 pandemic in Germany. BMC Public Health. (2022) 22:903. doi: 10.1186/s12889-022-13341-3 35524252 PMC9073434

[48] Kallmen H. Manifest anxiety, general self-efficacy and locus of control as determinants of personal and general risk perception. J Risk Res. (2000) 3:111–20. doi: 10.1080/136698700376626

[49] SharmaMCatalanoHPNaharVKLingamVCJohnsonPFordMA. Applying Multi-Theory Model (MTM) of Health Behavior Change to Predict Water Consumption Instead of Sugar-Sweetened Beverages. Journal of research in health sciences. (2017) 17(1):e00370.28413165

